# Danish translation and pilot testing of the European Organization for Research and Treatment of Cancer QLQ-TC 26 (EORTC QLQ-TC26) questionnaire to assess health-related quality of life in patients with testicular cancer

**DOI:** 10.1186/s12955-018-0954-3

**Published:** 2018-06-18

**Authors:** Louise Bager, Abbey Elsbernd, Aase Nissen, Gedske Daugaard, Helle Pappot

**Affiliations:** 1 Department of Oncology, Rigshospitalet, University Hospital of Copenhagen, Blegdamsvej 9, DK-2100 Copenhagen, Denmark; 2 University of Kansas School of Medicine, Kansas City, USA; 3 Documentation and Quality, Danish Cancer Society, Copenhagen, Denmark

**Keywords:** Testicular cancer, HRQoL, TC26, EORTC, Quality of life, Urology

## Abstract

**Background:**

The European Organization for Research and Treatment of Cancer (EORTC) QLQ-C30 is a core questionnaire designed to evaluate health-related quality of life (HRQoL) of cancer patients participating in international clinical trials. It is available in several languages including Danish. The EORTC QLQ-TC26 is a supplemental module developed for patients with testicular cancer, which can be useful in clinical trials. Despite Denmark holding a high prevalence and incidence of testicular cancer, no Danish translation was previously available. This paper describes the translation process and pilot testing of the Danish translation of QLQ-TC26.

**Methods:**

The English language EORTC QLQ-TC26 was translated into Danish using forward and backward procedures with reconciliation. The translated instrument was assessed in semi structured cognitive interviews in a sample of 10 patients ages 20–56 receiving treatment for testicular cancer.

**Results:**

In one round of pilot testing, no changes were required for the Danish translation based upon patient comments. The Danish translation was agreed by participants to be both culturally acceptable and semantically comprehensible.

**Conclusions:**

The pilot testing of the Danish translation of the EORTC QLQ-TC26 was performed in one round of patient interviews; these results support the Danish translation as a comparable instrument to the English language version. However, further validation is required to ensure complete equivalency. These results support the use of the EORTC QLQ-TC26 in future clinical trials conducted with Danish-speaking patients.

## Background

Testicular cancer (TC) is the most common cancer in men between the ages of 15–45 years [1]. During and after treatment, some TC patients and survivors may face detrimental complications, including anxiety, depression, fatigue, infertility and sexual dysfunction [2, 3], while others report health-related quality of life (HRQoL) values similar to the general population when using EORTC HRQoL measuring instruments [3, 4]. However, these prior findings have not evaluated the specific features unique to TC, and many individuals experience lowered HRQoL as result of illness or treatment [5].

The European Organization for Research and Treatment of Cancer QLQ-C30 (EORTC) is a widely-utilized core questionnaire designed to evaluate HRQoL in cancer patients participating in international clinical trials [6–8]. It is the inherent design of the QLQ-C30 to serve as an all-encompassing keystone for the measurement of HRQoL; this instrument may be supplemented with validated distinct subscales for specific diagnoses and patient populations for more nuanced results [9]. The EORTC QLQ-TC26 is a supplementary module for the EORTC QLQ-C30 for the assessment of HRQoL in TC patients and TC-specific symptoms currently in Phase IV international field testing [10]. This instrument consists of 26 questions addressing quality of life within the following subscales: treatment side effects, treatment satisfaction, future perspective, job problems, family problems, infertility, communication, body image problems, sexual activity, sexual problems, sexual enjoyment, and testicular implant satisfaction. Answers are selected based upon patient-reported severity or intensity of TC-specific symptoms on a four-point Likert scale from ‘not at all’ to ‘very much’, with a reference frame of 1 week [11]. This instrument is designed for the use of testicular cancer patients both receiving active treatment, and survivors of testicular cancer, making it a versatile tool for this population. The instrument was additionally designed to be used in coordination with the original EORTC QLQ-C30 30-item instrument for thorough HRQoL assessment; as such, the 26 items are numbered for usage after the EORTC QLQ-C30, as questions 31–56.

Currently, the EORTC QLQ-TC26 is available in several languages, including Dutch, English, German, Italian and Spanish [10]. However, this specific instrument is not available in a Danish translation. Denmark holds the second highest incidence and prevalence of testicular cancer in Europe [12, 13], and this population is easily followed due to the presence of a national testicular cancer registry [14]. As such, the testicular cancer population in Denmark is a valuable population for further studies on QoL. A Danish translation of the QLQ-TC26 would allow for comprehensive evaluation of HRQoL in TC patient populations, as well as encourage comparison and coordination between different HRQoL data resources.

The aim of this study was to develop and pilot test a Danish translation of the English language version of the EORTC QLQ-TC26 for inclusion in future clinical trials.

## Methods

### Translation

Research collaboration represented by the authors of this article was established between a Danish steering group and representatives from the EORTC Quality of Life Group to ensure appropriate methodology and translation protocol.

The English-language EORTC QLQ-TC26 was translated according to the protocol and guidelines outlined by the EORTC Quality of Life Group [15]. The translation was produced by a forward–backward procedure, in which two native Danish speakers with academic English translation experience independently translated the English EORTC QLQ-TC26 into Danish [15–17]. The two translations were compared by the steering group for any discrepancies. Consensus was achieved based on the following criteria: the translation should be in accordance with the original English text; Danish culture must be taken into account in choosing the right words and in construction of sentences; traditional standards are to be considered in terms of prior communication with patients and health care classification in response categories.

The compiled result was then back-translated by two native English speakers fluent in Danish with experience in academic Danish translation back into English. All translations, and a report detailing action thus far, were then sent to a representative from the EORTC Quality of Life Group, and were proofread by an independent translator. Results were described in an interim report, and then were assessed via pilot testing.

### Pilot testing

Pilot testing of the Danish translated version of EORTC QLQ-TC26 was performed by individual semi-structured interviews with 10 patients. This sample size was decided based on the recommended number of participants for translation pilot testing specified in the EORTC translation protocol [15]. The interviews were performed by a research nurse specialized in oncology. The interviews took place on the clinical ward after the patient’s consultation with an oncologist consecutively in the order they were scheduled in clinic for the relevant day. Inclusion criteria for patients were as follows: 1) A diagnosis of TC, 2) Ability to speak and read the Danish language, 3) At least one prior treatment with chemotherapy. Due to ethical concerns, patients who were about to receive a negative message (e.g. progression of disease) were not addressed for interview.

The patients were introduced to the project by the interviewer, and the aim of the project was described before the patient agreed to participate. Ten patients were invited to participate, of which all consented and agreed to participate. This research was exempt from review by an institutional review board or ethical authority under Danish law. Informed consent was obtained from all individual participants.

In the interview, the patients first filled in the questionnaire on their own and were asked to mark questions they found difficult or problematic. According to the topic guide for the interviews, the interviewer discussed each marked item on the questionnaire asking participants whether the translated item was: 1) difficult to answer; 2) confusing; 3) difficult to understand; or 4) upsetting/offensive. Participants were additionally asked how they would phrase the question in their own words. If a participant had any comments on the translated item, they were asked to rephrase the question in a way that would be less confusing, more easily understood, and/or less upsetting or offensive. The interviewer additionally asked the patients about their general thoughts related to answering the questions and what problems they had experienced in order to identify the reasoning behind their comments. The interviewer kept notes throughout the interview, summarized and repeated their notes for the patient before closing the interview.

### Data analysis

The steering group reviewed the results of all interviews, and made decisions based on all questions identified as problematic by participants.

Data analysis was performed according to the procedures used in the translation manual from the EORTC Quality of Life Group [15]. The steering committee compiled a summary of participant comments from pilot testing interviews. All items that elicited comments from participants were examined to determine the nature of the problem, and what suggestions were made for alternate phrasing. This report was then sent to the EORTC Quality of Life Group, and results were discussed both within the steering committee, and then with the EORTC Quality of Life Group. If changes were determined necessary, further re-testing would be performed.

### Compliance with ethical standards

#### Disclosure of interest

The authors declare that they have no conflicts of interest.

#### Ethical approval

All procedures performed in studies involving human participants were in accordance with the ethical standards of the institutional and/or national research committee and with the 1964 Helsinki declaration and its later amendments or comparable ethical standards. This research was exempt from review by an institutional review board or ethical authority under Danish law.

#### Informed consent

Informed consent was obtained from all individual participants included in the study.

## Results

The forward translation was performed by two native Danes with academic experience in translation of English texts with the Danish Cancer Society. In eight items full agreement was found in translation between both translators. In 16 items, minor differences were found in word choice, but there was no difference found in meaning. In 2 items, different words were utilized to describe symptom with slight alteration in interpretation, which were discussed to determine the most accurate statement in reconciliation with the English text. The following decisions were made: in item 39, ‘…the medical treatment…?’ was chosen to be utilized over ‘…the doctor’s treatment…?’, and in the introductory text ‘Patients sometimes report…’ was chosen to be utilized over ‘Patients sometimes tell…’.

The backwards translation was performed by two bilingual native English speakers, one originating from the USA and the other from the UK. Both have experience in translation of Danish texts with the Danish Cancer Society. In 10 items there was full agreement in translation between the two translations and in accordance with the original English text. In 15 items there were minor differences in word choice with no difference in meaning in comparison to the original English text. In one item, the backward translation resulted in a different meaning in comparison to the original English text. The backward translation item 24, ‘to what extent was sex enjoyable for you,’ resulted in ‘..was sex pleasant ..’ and ‘.. was sex comfortable ..’. This was considered not to reflect the original meaning. The exact Danish word for ‘enjoyable’ was chosen from one of the forward translations and changed in the question item. This was the only change made to the original Danish text, and no further changes were determined to be required from the translators.

Pilot test interviews lasted between 15 and 20 min each. Patients were between the ages of 20 and 56 with an average age of 41. Participant demographics are summarized in Table 1.

**Table 1:** Characteristics of patients

Age	Education	Civil status
56	Skilled worker	Married/couple
38	Higher education (3–4 years)	Single
42	Higher education (3–4 years)	Married/couple
20	Student	Single
52	Higher education (> = 5 years)	Married/couple
52	Skilled worker	Single
24	Higher education (3–4 years)	Single (girlfriend)
46	Higher education (3–4 years)	Married/couple
48	Higher education (3–4 years)	Married/couple
35	Primary school	Single

Interviews elicited 11 total comments on 8 different EORTC-TC26 questions by five distinct patients (50% of participants). Comments could be applied to multiple statement categories. Four comments were difficulties in understanding, six were confusing statements, and four were suggestions for alterations in wording. One statement requested clarification in time dimensions within the EORTC questionnaire as a whole. Table 2 summarizes the comments for each question and patient. There were no comments related to difficult words or upsetting questions.

**Table 2:** Comments obtained for each question and patient

Question nr.	Difficulty understanding	Confusing	Suggested wording
31		A little confusing as you can lose hair as a man regardless connection to cancer	Have you lost hair in relation to your treatment?
32		Is it only interesting if it is experienced as a problem? What if you experience a change but not think of it as a problem	
38		A little confusing only to ask about hearing badly, what about tinnitus – it is important – but will it be covered by this question?	
39	Wording of the time dimension. Shall it be understood for the past or the present time?		
40	*(As in question 39)*		
42	*(As in question 39)*		
45	Difficult to understand what the question is about		
		The word ‘disruption’ seems wrong. Think ‘affect’ would be more appropriate	Were you worried about how it will affect your family life?
49		Confusing wording regarding the ‘time’	To what degree have you been interested in …?
50		Confusing wording regarding the ‘time’	To what degree have you been interested in …?
Other Comments		Is it relevant to relate answers to the time dimension” in the past week” and the grammatically framing of the time dimension in the questions?	

The steering group discussed the results from the 10 interviews and determined that no changes were required after discussion. Hair loss and problems with taste or sense of smell were discussed as items that could be either determined as unproblematic or unrelated to cancer treatment. Discussion between the steering group determined that changes in description would change the inherent meaning of these statements, and that patients will generally complete this questionnaire within the context of cancer treatment and not external causes. Tinnitus was mentioned as a symptom that may not adequately be covered, however, the steering group believed it was well-covered under the questionnaire item ‘problems with hearing’. Lastly, the word ‘disruption’ was discussed as a potentially inappropriate word choice, however, no other Danish word was available that could correctly be translated backwards. All justifications are summarized in Table 3. The final Danish translation has since been submitted to the EORTC Quality of Life Group.

**Table 3:** Concerns and discussion from steering group justifying changing or retaining current translation in question

Question Nr. / Topic	Concern	Discussion
Question 31 - Hair Loss	Men may lose hair for reasons not attributable to cancer.	Although the comment is understandable, it is of no significance for the question. Generally, the patient shall not consider the reason for the symptom. *No change is required.*
Question 32 - Problems with Taste or Sense of Smell	Problems with taste or sense of smell may be noted as changes that are not necessarily problematic.	The patient’s reflection on the symptom being a problem or not can be relevant. However, changing the word would lead to an incorrect meaning when compared to the original English text. *No change is required.*
Question 38 - Tinnitus	Problems with hearing may not adequately cover tinnitus.	We consider that ‘tinnitus’ would be captured in the question of ‘problems with hearing’ as most patients would consider it a problem. *No change is required.*
Questions 39, 40, 42, 49, 50 - Time Dimension	It is unclear if the statements are worded in past or present tense.	Two of the patients commented on the different wordings of the time ‘were you …’ or ‘have you been …’. Also, there was consideration if satisfaction with the medical care should be related to the past or the present time. We have decided not to make any change in the wording, as the shifts in wording follow the original English text. *No change is required.*
Question 45 - Disruption	Affect may be a more appropriate word choice than disruption.	Two patients commented on the wording of the question especially concerning the word ‘disruption’ and one suggested to use ‘affect’ instead. However, ‘affect’ would not be translated backwards to ‘disruption’. We have no other Danish word that would match ‘disruption’ translated as ‘forstyrrelse’ which was correctly translated backwards. *No change is required.*

## Discussion

In this paper, we have outlined the process of translating and pilot testing a Danish language translation of the EORTC QLQ-TC26 QoL instrument utilizing the EORTC Quality of Life Group translation procedure [15]. The end result translation was consistent with the EORTC-TC26 English language version. This pilot testing of the Danish translation of EORTC QLQ-CT26 was performed in one round of patient interviews with no changes required in the questionnaire’s wording. The EORTC QLQ-TC26 Danish translation is now available for use in cancer clinical trials through collaborative agreements with the EORTC Quality of Life Group.

Strengths of this translation and pilot test include utilization of the official translation procedure for an EORTC instrument [10, 15]. While a small population (*n* = 10) was identified from a single site, the criteria have been met for an official standardized EORTC translation pilot test [15]. This pilot testing protocol is limited as interviews were not recorded and transcribed verbatim. However, the interviewer completed thorough notes, and repeated the contents of these notes to participants, ensuring accuracy of interview content and meaning throughout the pilot testing process.

There has been prior commentary about the limitations of the EORTC QLQ-TC26 as a new instrument [4], however, with more time and sufficient use, the EORTC QLQ-TC26 will be better able to be assessed for utility in testicular-cancer specific QoL measurement. Phase IV field testing for the EORTC QLQ-TC26 is currently underway to assess for dimensionality, reliability, sensitivity to change and instrument validity [10].

## Conclusions

In conclusion, the pilot testing of the Danish translation of the EORTC QLQ-TC26 was performed in one round of patient interviews; these results support the Danish translation as a comparable instrument to the English language version. The EORTC QLQ-TC26 has begun to see use in clinical trials [EudraCT2014–003930-17][NCT02304575], and the results of this translation and pilot testing process provide preliminary support for the use of the EORTC QLQ-TC26 Danish translation in testicular cancer clinical trials. Next steps will include the utilization of this QoL tool in clinical trials with testicular cancer patients amongst a Danish-speaking population, as well as validation of this translation in a larger population of Danish-speaking patients.

## Abbreviations

EORTC: European Organization for Research and Treatment of Cancer
HRQoL: Health-Related Quality of Life
TC: Testicular Cancer
